# Exploring the Biological Activities of Ionic Liquids and Their Potential to Develop Novel Vaccine Adjuvants

**DOI:** 10.3390/vaccines13040365

**Published:** 2025-03-28

**Authors:** Snehitha Akkineni, Mutasem Rawas-Qalaji, Samir A. Kouzi, Christiane Chbib, Mohammad N. Uddin

**Affiliations:** 1 College of Pharmacy, Mercer University, Atlanta, GA 30341, USA; snehitha.akkineni@live.mercer.edu; 2 College of Pharmacy, University of Sharjah, Sharjah 27272, United Arab Emirates; mqalaji@sharjah.ac.ae; 3 School of Pharmacy, Wingate University, Wingate, NC 28174, USA; skouzi@wingate.edu; 4 College of Pharmacy, Larkin University, Miami, FL 33169, USA

**Keywords:** ionic liquids, adjuvants, vaccines, antigens, immune response

## Abstract

Ionic liquids (ILs) are salts with poorly coordinated ions, allowing them to exist in a liquid phase below 100 °C or at room temperature. Therefore, they are best described as room temperature ionic liquids (RTILs). In ionic liquids, the presence of a delocalized charge in at least one ion, coupled with an organic component, inhibits the establishment of a stable solid crystal lattice. Due to their flexible properties and several distinctive characteristics, such as high ionic conductivity, high solvation power, thermal stability, low volatility, and recyclability, ILs have been extensively used in chemical industries. In addition to their various other applications, they also hold potential for drug formulation development. Ionic liquids can be used as solubility enhancers, permeability enhancers, stabilizers, targeted delivery inducers, stealth property providers, or bioavailability enhancers. Moreover, ILs hold significant potential in vaccine formulation. Many new vaccines are in the pipeline with different types of antigens; however, the existence of only a limited number of adjuvants hinder their potential use. Thus, developing new, highly effective, low-cost adjuvant preparations is a central interest among formulation scientists. With their unique properties and biological functions, ILs can be highly promising candidates for new types of vaccines.

## 1. Introduction

**Figure 1:**
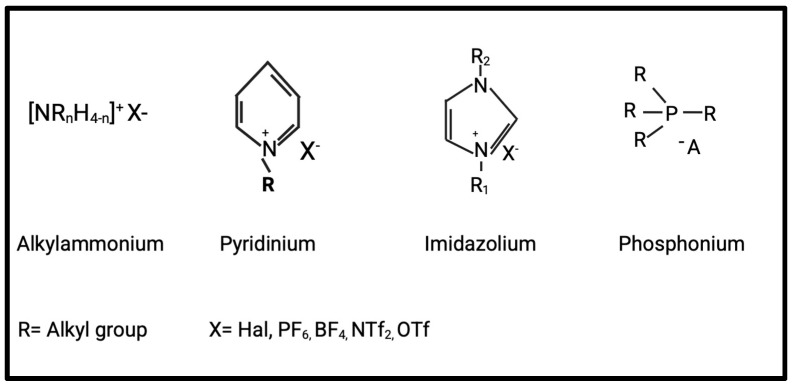
The three main types of ionic liquids.

Ionic liquids (ILs) have recently attracted interest due to their safety and versatility in various applications. ILs, generally composed of organic cations and organic/inorganic anions, are a class of molten salts with melting points below 100 °C. The cations are mostly alkylated imidazole, pyrrole, or pyridine derivatives, quaternary alkyl amines, and alkyl phosphines. Representative anions include halides, alkyl sulfates, fluorinated hydrocarbons, carboxylic acids, and amino acids [1]. Ionic liquids possess distinctive characteristics that have enabled their use across various research fields. They are nonvolatile, nonflammable, and recyclable components with excellent solvating power, good thermal stability, and tunable properties via the use of appropriate cations and anions [2]. Another advantage of ionic liquids is that altering the cations and anions allows for the adjustment of their physical and chemical properties, such as solubility, polarity, viscosity, and acidity, to meet specific needs. Due to this flexibility, ILs have been applied in processes such as catalytic reactions, desulfurization, hydrogenation, and electrochemistry [3]. ILs are broadly classified into four types depending on the cations: (1) alkylammonium, (2) dialkylimidazolium, (3) phosphonium, and (4) N-alkylpyridinium-based ILs [4] (Figure 1).

**Figure 2:**
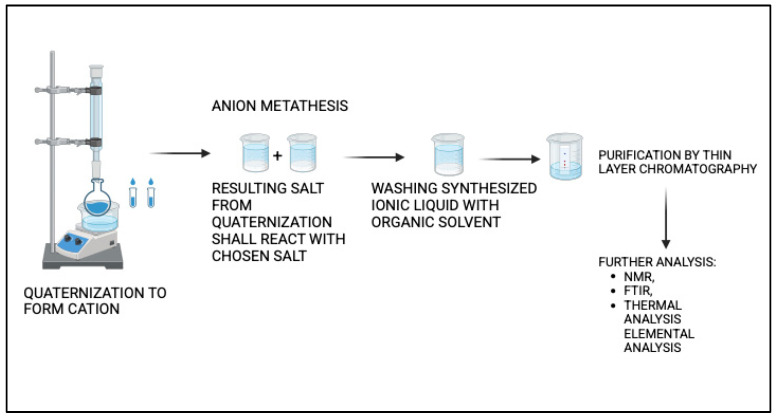
A general schematic representation of the synthesis and purification of ionic liquids.

Ionic liquids (ILs) have gained significant attention in pharmaceutical and biomedical research due to their unique physicochemical properties of low volatility, high thermal stability, and tunable solubility. Solubility assessment is an important pre-clinical study for any drug in the pharmaceutical industry. Ionic liquids are potential solubility enhancers for drugs. A drug must be soluble in body fluids to reach the site of action. For a drug to be effective, it must be soluble enough to be absorbed into the bloodstream. Solubility affects all pharmacokinetic stages of absorption, distribution, metabolism, and excretion (ADME). A drug with poor solubility may have unpredictable pharmacokinetics, leading to variable therapeutic outcomes. Poor solubility can lead to low bioavailability, meaning less of the drug reaches the target site [5]. Several studies have shown that ionic liquids can enhance the solubility of a drug when used with optimized anions and cations [6,7,8]. A generalized method for the synthesis of ionic liquids is shown in Figure 1, and the structural formulas for some of the ILs are shown in Figure 2.

ILs also have potential for use as drug stabilizers. ILs can stabilize active pharmaceutical ingredients (APIs) by protecting them from degradation caused by environmental factors such as light, heat, and moisture. Ionic liquids can be used to create formulations that allow for controlled release of the drug, ensuring a steady and prolonged therapeutic effect. ILs can also stabilize drugs by preventing crystal formation, one of the major issues in the drug industry. Preventing crystallization is important for maintaining the drug’s stability and bioavailability. ILs can be used in various drug forms, including liquids, gels, and solid dispersions, providing flexibility in drug formulation. IL-based stabilizers may help keep vaccine formulations unchanged when exposed to heat, light, acidity, or humidity. Such stabilizers also help vaccine antigens, such as proteins, peptides, enzymes, inactivated bacteria, viruses, and virus-like particles, to stay in their original forms [9,10]. They exhibit better permeability and bioavailability when used with acidic and basic drugs [11]. Chemical companies like Arkema have patented the production processes for high purity ionic liquids such as the imidazolium-based ionic liquids made by Proionic’s through the patented Carbonate-Based Ionic Liquid Synthesis (CBILS) process.

Ionic liquids can also act as permeability enhancers in various drugs. Permeability is a critical factor for many dosage forms, such as buccal, sublingual, and transdermal patches, which need to bypass the first-pass effect for rapid onset of action. Kouzi et al. studied the ionic liquid 1,4-diazabicyclo [2.2.2] octane (DABCO) as a permeability enhancer in the buccal administration of a diphenhydramine hydrochloride drug in quickly soluble oral dissolving film. The results of this study showed that film dosage forms with the ionic liquid DABCO are capable of enhancing not only the permeability of the drug but also the release of the drug from the film [12].

Ionic liquids (ILs) (Table 1) hold great promise for various applications in pharmaceutical formulation development, particularly as vaccine adjuvants. Adjuvants are essential components of modern vaccines [13]. They play a crucial role in the following ways: (a) enhancing the ability of vaccines to elicit a strong and durable immune response, even in immunocompromised individuals such as neonates, the elderly, and immunosuppressed patients; (b) reducing the required antigen dose and number of immunizations; and (c) modulating the nature of the immune response [14]. As adjuvants, ILs can improve drug delivery, stabilize biomolecules, and enhance therapeutic efficacy. Understanding their interactions with protein targets is vital for optimizing their biomedical applications. Despite the increasing demand for new adjuvants to boost vaccine efficacy, only a few have been successfully used in vaccines [15]. Current adjuvants like alum (aluminum salts) and MF59 have been in use for a long time but have limitations in stimulating robust cellular immunity, which is crucial for combating pathogens in diseases [16]. The development and regulatory approval of new adjuvants face numerous challenges, including extensive safety and efficacy testing, high development costs, and the complex nature of immune system modulation [17]. Many potential adjuvants are in the preclinical stage, but their progression to approved products is hindered by strict regulatory requirements, incomplete understanding of their mechanisms of action, lengthy testing timelines, and the difficulty in demonstrating clear benefits over existing options, especially when switching between different vaccines [18]. Additionally, adjuvants under research face intellectual property (IP) and freedom-to-operate (FTO) constraints. With the IP/FTO landscape for proven adjuvants now more open, developing innovative adjuvant formulations and antigen designs has become a key strategy in vaccine development. Therefore, there is an urgent need for new adjuvants that can address these issues in vaccine formulations. The safety, solubility, flexibility, and tunable properties of ionic liquids make them promising candidates as adjuvants. In this review article, we explore the use of ionic liquids as vaccine adjuvants, evaluating their biological activity, identifying potential target molecules that can trigger immunogenicity induced by ionic liquids, and assessing their immunogenicity with different types of antigens.

## 2. Perspectives and Challenges of Ionic Liquids

With their excellent properties, ILs’ applications are widespread across several industries. ILs are used in polymer technology serving as solvents for both polymer synthesis and depolymerization. For example, 1-butyl-3-methylimidazole chloride is employed as the ionic solvent to reduce the polymerization of larix bark as a source of UV blocking for cosmetics [19].

ILs are also well known for their applications in the biomedical field due to their prominent potential to improve the solubility of drugs, reduce crystal instability, and also enhance drug efficiency [20]. However, there has been a significant shift in perception from the initial observations that ILs are nonflammable and are stable in water to current observations stating that some ILs are flammable and unstable [21]. One of the major advantages of ILs is that they can be fully recoverable and reusable, but choosing the appropriate IL is extremely important as they might have interactions with the polymer in terms of their melting point and solubility [22]. The current research is focusing on making ILs commercially feasible and expanding their applications in pharmaceuticals and energy storage. Amongst their several applications, this study discusses the potential of ILs as adjuvants in later sections.

Contrary to their advantages, ILs also face numerous challenges in their widespread applications. The cost of the ILs is one of the major drawbacks; though they can be synthesized relatively easily, most of them are comparatively more expensive than conventional solvents. According to the total annualized cost, some ILs such as triethylammonium can be produced for the lowest cost of USD 1.24 kg^−1^, but this is not the case for all other ILs, and they can get expensive [23]. Furthermore, because ILs are not produced on a large scale, their availability also poses a concern that could hinder research and development. Although popularly termed as ‘green solvents’, ILs cannot entirely be considered as environmentally friendly compounds. Due to their high thermal stability, ILs turn non-volatile in room temperatures, and their wide application range is now a posed threat to aquatic and terrestrial environments.

Moreover, the synthesis and additional purification of ILs is comparatively more expensive than regular solvents, and their viscosity is considerably high, limiting efficient mass transfer, thereby hindering the industrial processes [24]. Since ILs are used as stabilizers, and given their ability to sustain higher temperatures, knowing their thermal and electrochemical properties before their application is necessary [25]. A lack of standardization and guidelines for ILs renders it challenging to ensure that they are safely handled, stored, and disposed. The toxicity of the ILs, in general, as well as their effects on microorganisms and other aquatic organisms, is explained in detail in the toxicity section. More research pertaining to ILs’ environmental impact is necessary; even though they cause low pollution compared to other solvents, they are found in high levels in the aquatic systems and in soil. ILs could also be found in deeper soil layers, contaminating the groundwater. Some lipophilic ILs also sorb into the soil, with clay particles in the soil being more prone towards higher sorption [26]. Anions are not retained in the soil, unlike imidazolium and pyridinium IL cations which are adsorbed in the soil [27]. Moreover, ILs can have a long half-life up to 670 days, suggesting their persistence in the ecosystem and their resulting risk [28]. ILs also undergo several environmental transformations like photochemical reactions where they interact with hydroxyl radicals (-OH) and triplet state dissolved organic matter which contribute to their transformation, and they might also be slowly broken down by certain environmental microorganisms despite their resistance towards biodegradation [28,29]. 1-Octyl-3-methylimidazolium (C8mim) was found to be present in the environment and is one of the triggers for primary biliary cholangitis, playing a major role in autoantigen exposure [30]. Furthermore, the environmental fate of the ILs are closely linked to their bioaccumulation and toxicity, and aquatic ecosystems are highly affected due to the mortality of organisms that results and the interactions of ILs with aquatic species [29].

To minimize these effects, ILs are sometimes reused after certain cycles, which may change the ecotoxicity and improve their sustainability [31]. Several methods exist to recycle ILs, one of which involves destroying the IL and removing the contaminants through photolytic degradation [32,33]. The practical applications can be improved through efficient recycling of ILs and catalysts by nano-filtration [34]. However, for hydrophobic and hydrophilic ILs, the recycling comes with one disadvantage in that they cannot be used for industrial purposes, and membrane separation techniques can be used to overcome this [35].

## 3. Biological Activities of Ionic Liquids

With their diverse properties, including their biological activity, ionic liquids are known to be used for a wide range of applications. As they possess high solubility, they can dissolve both organic and inorganic compounds and are also proven to have interactions with proteins. Studies have shown that ILs have the capability to dissolve the proteins that are generally insoluble in other organic solvents [36]. Over the decades, there has been an increase in research regarding the usage of ILs for protein dissolution and stabilization. When studying the molecular packing of proteins, it is important to consider several factors like the proteins’ structure and their solubility in ILs. Despite ILs having similar solubility and polarity to proteins, they can only dissolve certain ones like lipase and cellulase [37]. The presence of hydrogen bonds through anions makes it easier for the ILs to interact with proteins [38]. Additionally, protein stability can be achieved through a balance of stabilizing and destabilizing forces. As surface-active ionic liquids (SAILs) have both hydrophilic and hydrophobic functional groups, they can be used to enhance the protein solubility and further be encapsulated for drug delivery. Considering the presence of both functional groups, SAILs could interact with different areas on the protein providing them the opportunity to either stabilize or destabilize it. However, the disruption or stabilization will only occur based on the protein’s structure and its concentration [39]. Based on a recent study on Ubiquitin, the authors have found that Ubiquitin stability was dependent on the hydrophobic interactions, and the anion–cation variations in the IL also influence the interactions with proteins. Moreover, the longer alkyl chain containing cations with great hydrophobicity (1-Butyl-3-methylimidazolium[BMIM]+ >1-Butyl-1-methylpyrrolidinium [BMPyr]+ > 1-Ethyl-3-methylimidazolium [EMIM]+) have shown an increased destabilization effect on Ubiquitin while the anions followed the Hofmeister series order [40].

ILs tunability and their nature makes them great solvents, and when utilized as solvent medium they have notable impacts on enzyme stability. The anion part of the imidazolium-based ILs plays a major role in altering their effects on enzymes. It is observed that the enzymes are stable in lower IL concentrations than higher concentrations which may cause destabilization or deactivation [41]. Kacem S.H. et al. have studied the effect of 10 newly synthesized ILs on the laccase enzyme. Of the 10 ILs, 1-Butyl-3-methylimidazolium acetate [BMIM][Ac], Choline Acetate [Chol][Ac], and Choline propionate [Chol][Prop] were shown to improve enzyme activity and stability. Despite showing a 20-fold increase in laccase activity, [BMIM][Ac] did not protect the enzymes against high temperatures and showed no effect on long term preservation. With the [BMIM][Ac] being adsorbed on the enzyme surface, there is less substrate binding, and it was observed that the enzyme activity was increased by the ILs by increasing the catalytic markers Vm and Kcat [42].

ILs were used to solubilize the P450 decarboxylase enzyme by Nicholson J H et al., and the results show that the enzyme stability enhanced the substrate solubility, thereby proving that the ILs can be potentially used as biocatalytic process enhancers [43]. Another study by Ji L et al. has shown that the amino-functionalized ionic liquid (NIL)-modified metal–organic framework (UiO-66-NH_2_) can be used to immobilize Candida rugosa lipase (CRL) by using Dialdehyde starch (DAS) as a crosslinker. It was observed that, due to the covalent bonds between (UiO-66-NH_2_) and DAS, the carrier lipase strength was increased, thereby enhancing the catalytic activity. Although the presence of ILs has shown a reduction in specific surface area, the immobilized CRL still had excellent catalytic activity, proving the ILs’ potential in enzyme immobilization [44].

All the above findings demonstrate the ILs’ ability to enhance enzyme activity while also showing their potential as novel biocatalyst enhancers. Beyond the proteins and enzymes, ILs have also been studied to understand interactions with genetic material, particularly DNA.

The biological activity of ILs on DNA has shown that they can be used to extract specific DNA sequences, and current research has provided several insights on their interactions. Khavani M. et al. have studied the DNA and amino acid ionic liquid interactions to understand the long-term stability of DNA during storage. In the presence of 1-butyl-3-methylimidazolium glycinate [BMIM][Gly], the DNA exhibited its maximum stability and highest interactions when compared with other ionic liquids and water. Moreover, it was studied that the IL-DNA complex formation is mainly due to the molecular orbital interactions along with having a more stable structure in IL–water mixtures [45].

Tulsiyan K D. et al. have performed docking studies to understand the molecular mechanisms of ILs interacting with DNA. UV-vis spectra and molecular docking have confirmed the hydrophobic interactions, hydrogen bonding, and electrostatic forces that play a major role in the IL-DNA interactions. The magnetic ionic liquids used in the study are [Ch][Fe] and [Ch]_2_[Mn]; out of the two, [Ch]_2_[Mn] demonstrated a notable shift in DNA, i.e., it exhibits increased DNA curvature and compaction compared to its initial form. The stronger affinity of [Ch]_2_[Mn] with DNA is further confirmed by the high binding free energy ΔG observed at −642 kcal mol^−1^ [46]. However, recent studies have shown that the hydrated ILs such as Choline dihydrogen phosphate (DHP) ensure the stability of biomolecules, with a DNA triplex formation in these. And the absence of nuclease ensures the long-term stability [47]. Contrary to these reports, there has been DNA damage for cells when combined with ionic liquids, such as inducing DNA fragmentation for multiple human cell lines [48,49].

With the development of API-ILs, the compounds were found to bind with the cell membrane better with an improvement in the hydrophilicity when the compounds were connected to the cations of ionic liquids [50]. ILs also showed promising results as antimicrobial agents using their electrostatic forces to settle into the membrane and have also been used with other materials to develop beneficial anti-microbial formulations, causing DNA conformational changes leading to the prevention of RNA messenger transcription [50,51].

## 4. Toxicity and Fate of Ionic Liquids

Despite the extensive use of ionic liquids over the years, their toxicity remains a concern that needs to be addressed. The toxicity of the ILs is primarily influenced by their structural component, i.e., their cation structure, with the increase in hydrophobicity and length of the side chain leading to an increase in toxicity [52]. However, among the three generations of ILs, the third generation Cholinium-based ionic liquids have been determined to have the lowest toxicity followed by piperidinium, pyridinium ILs, and imidazolium-based IL’s having the highest toxicity [53]. With the advantageous tunability of the ionic liquids, alkyl chains can be easily modified by adding functional groups, causing changes in the physicochemical properties. However, several studies have reported that the increase in the alkyl chain length significantly increases the toxicity as there will be enhanced cell membrane interaction due to lipophilicity [54]. The toxic effects of the ILs are exhibited through several mechanisms, primarily with the amphiphilic nature of the ionic liquids, which aids in their binding to cell membranes, and as their binding increases, the membrane stability decreases, leading to cell death [55]. Moreover, imidazolium- and pyridinium-based ionic liquids can inhibit enzymes, specifically acetylcholinesterase, causing a severe disruption in neurological processes. The structural attributes, specifically the alkyl chain length and cation type, influence the inhibition of acetylcholinesterase, which further leads to the degradation of the neurotransmitter acetylcholine [56]. To overcome the toxicity, structural modifications like using cations with short alkyl chain lengths and the introduction of polar groups like hydroxyl or ether groups to the cation can be considered [57]. Design eco-friendly ionic liquid structures using quantitative structure–activity relationship models will help in providing toxicity estimations [58]. Using machine learning datasets with preidentified patterns helps to establish the structure–activity relationships, allowing the researcher to understand the toxicity profile and choose the ionic liquids accordingly [59].

As for the ILs’ fate in animal and human bodies, it has been established that they interact with the biological components through cell membrane interactions and antimicrobial activity and possess biocompatibility. Initially, they are absorbed into the bloodstream through cation transporters, and the efflux and absorptions behaviors are less dependent on cations. Once distributed, they have a quicker excretion through urine and feces [60]. Hattori et al. used Choline and geranic acid to assess the transdermal delivery of Nobiletin, proving that ionic liquids enhance penetration, thus improving its bioavailability [61]. This result proves their ability to overcome biological barriers, improving both oral and transdermal delivery. Apart from these, ILs are also known for their cell interactions and their alterations using various methods such as lipid distribution and membrane permeabilization. They also play a major role in the damage of chloroplasts in plants and could interfere with the signaling pathways, thus causing the fragmentation of DNA [62]. Moreover, they also have the ability to interact with the cell membrane and disrupt it, thus altering the permeability [63]. The major factors affecting the fate of the ILs would be their structure and concentration. As the cations and anions interact differently with biological systems, their toxicity will be varied; similarly, the effect of ILs on the human body depends upon the concentration of the IL. Several studies were conducted using higher concentrations of ILs up to 100 mM, leading to cell death [64]. Furthermore, ILs can also enhance the level of reactive oxygen species (ROS), which lead to oxidative damage to the cells along with lipid peroxidation and protein damage. Huiyang fan and Huijun et al. have studied different ionic liquid anions’ and cations’ role in Scenedesmus obliquus and have found that ILs induce dose-dependent toxicity. They noted that the toxicity was highly dependent on the time of exposure, where both anions and cations are contributing to the toxicity. However, the effects of cations were more obvious in the imidazolium-based ILs when compared to pyridinium-based ILs in algae. The high toxicity is said to be due to the enhanced ROS levels, and this excessive ROS production is elicited by the exposure to ILs, making them the major contributors towards toxicity. These high ROS levels have also caused growth suppression and membrane damage leading to cell death [65]. ILs were also studied for their toxicity against microorganisms and marine organisms. Several ILs, including 1-Butyl-3-methylimidazolium bromide [BMIM][Br] and 1-Hexyl-3-methylimidazolium bromide [HMIM][Br], were observed to have toxicity in Vibrio fisheri, and this toxicity was increased with an increase in the alkyl chain length [66]. In another study, imidazolium, pyridinium, and ammonium compounds were studied for toxicity in Vibrio fischeri and daphnia magna. The concentrations ranged from 0.001 to 100 mmol L^−1^, with ammonium exhibiting the least toxicity followed by pyridinium and imidazolium [67]. Additionally, 1-butyl-3-methylimidazolium chloride[BMIM]Cl was studied in saccharomyces cerevisiae; though the cell morphology was not changed, the reproduction rate was observed to decrease with an increase in the concentration of IL, and the IC_50_ value was 0.39 gL^−1^ [68]. In another study using imidazolium and pyridinium ionic liquids, toxicity was assayed using agar diffusion in Escherichia coli and Bacillus subtilis; the bacterial inhibition halo was higher in B.subtilis than E.coli when exposed to ILs [69]. [HMIM][Br] in daphnia magna has shown that, when exposed to ILs, the survival was reduced along with a decline in the offspring’s survival rate [70].

## 5. Potential Immunogenic Targets of Ionic Liquids

Immunogenicity is the ability of molecules to generate immune responses. Ionic liquids are best known for interacting with cell membranes due to their amphiphilic nature; having a cation attached to the long alkyl chain explains this. Due to ILs’ nonpolar nature, the cations usually remain at the polar surface allowing the ILs to interact with cell membranes causing alterations within proteins and thus exposing any hidden epitopes [71]. Klahn et al. have shown that a cation, acyclicbutylpentamethylguanidinium-([BAGUA]^+^), when interacting with CAL-B protein, undergoes destabilization because of conformational changes, thus exposing the protein core to the ionic liquids and, hence, stabilizing the unfolded protein [72]. Considering this, there is the possibility of linking the cellular functions of ionic liquids to prompt an immune response, particularly when combined with toll-like receptors and scavenger receptors. As studied by Dhiman D et al., Cholinium ILs have been used to stabilize Immunoglobulin G, providing both conformational and thermal stability. The stability in aqueous solutions was assessed through spectroscopic and light scattering techniques [73]. The study suggests that these ILs can also further be explored to stabilize other antibodies. Given this mechanism, their potential role in enhancing immunogenicity could be explored as they might influence antigen presentation.

TLRs bridge the link between the innate and adaptive immune system through the activation of antigen-presenting cells and by simulating cytokine production. Additionally, they also regulate T cell activation for adaptive immune responses [74]. Moreover, the scavenger receptors play a crucial role in innate immunity by recognizing diverse array of ligands, including PAMPs. Having this ability makes them a key regulator in immune homeostasis [75]. Ionic liquids can be strategically combined with TLR agonists and scavenger receptors to enhance immune activation, facilitating targeted immune responses. In a study by Shimizu et al., Wilms tumor 1 peptide, OVA peptide, and Resquimod (R848), a toll-like receptor 7 agonist, were combined with Isosteric acid and tartaric acid along with other excipients to develop three formulations. One formulation included both the peptide and R848, the second only featured the peptide, and the third only featured R848. The IL-based formulation of 20 mg was applied to each mouse with a nonwoven fabric/PET film and was fixed for 24 h with tape. Then, 6–8-week-old C57BL/6N mice were subcutaneously injected with LLC 5 × 10^5^ or EG7-OVA 1 × 10^6^ cells. Skin irritation due to IL-based treatments was evaluated to study immunization-related skin toxicity. After the development of the tumor, the formulations containing WT1 peptide, R848, OVA peptide, or WT1 long peptide were individually applied to the abdominal skin for 24 h and removed the following day. The IL-based sequential immunizations strongly suppressed the tumor growth when compared to non-treatment groups, validating its efficacy as a vaccine therapy. Despite the tumor suppression, the formulation resulted in temporary skin inflammation, and the individual effects of APIs have not been validated in the current study for antitumor immune responses. Additionally, there was no induction of skin irritation due to the sequential immunization of IL-based formulations. The study showed a higher permeability of the peptides with IL and reduced tumor growth in a murine model, thus suggesting increased immune responses [76].

There are studies that showed improved immune responses by incorporating ionic liquids in their formulations. In a study, Choline and Niacin (ChoNic) were formulated as an oil in an ionic liquid nano emulsion-based adjuvant formulation. This formulation was compared with an unadjuvanted iFMDV and with Montanide ISA 206-adjuvated iFMDV. To determine the ILs’ efficacy in generating immune response, the iFMDV-specific IgG antibody responses were measured in 6–8-week-old female BALB C mice that were subcutaneously immunized twice with all the groups. Following the first immunization, the responses were measured at 14 days, and the IL adjuvanted formulation exhibited 2.8-fold higher titers than unadjuvanted iFMDV and 2-fold higher titers than the o/w emulsion. These were increased to 4-fold and 3-fold higher titers after 14 days of booster immunization, respectively. Interestingly, there were no major differences when compared to the Montanide ISA 206 group. The IL formulation also showed higher potential to induce the iFMDV-specific IgG titers. The interleukin 4 section corresponds to the TH2 humoral response, and interferon gammas corresponds to the Th1 cellular responses [77]. And both responses were considerably higher in the IL group than that of the o/w and PBS groups. Montanide ISA 206 has shown lower activated B lymphocytes than the IL group, yet there was no difference with the IgG serum titers. These results summarize that the ChoNic-based oil in the IL formulation has successfully demonstrated the elicitation of immune responses for iFMDV [78]. Though there were no adverse effects or cytotoxicity concerns observed in the experimental model, the cytotoxicity of the nano emulsion itself has not been reported, which would have been beneficial.

In another study, Cholinium dihydrogen phosphate [Cho][H_2_PO_4_], Cholinium Chloride [Cho][Cl] and Cholinium Sulfate [Cho][SO_4_] were used to formulate 0.5 M solutions with 146S antigen adjuvanted with Montanide ISA 206, and Balb/C mice were subcutaneously immunized using 12S in PBS as the negative control. Although there were no significant improvements in antigen titers, ChoSO_4_ and ChoCl showed minimal improvement; the animal studies revealed that the antigens did not attenuate with all the three ILs tested. However, a significant increase in the half-life of the iFMDV virus for long-term storage was observed, outperforming 10% *w*/*v* sorbitol and 10% *w*/*v* sucrose. The study also stated the importance of choosing the appropriate anion selection in ionic liquids, as ChoH_2_PO_4_ has shown a destabilizing effect on iFMDV particles [79].

Moreover, ionic liquids were also used to enhance the antigen delivery through the transdermal route. A transdermal patch was prepared using an IL-based solid in an oil nano dispersion along with a pressure-sensitive adhesive for the delivery of the macromolecular antigenic protein ovalbumin (OVA). Amongst the previously developed IL formulations for transdermal delivery, the major disadvantage is the low viscosity; the current rationale of the study is to address this issue through a patch-based ionic liquid in an oil formulation. The patch was set in a pressure-based adhesive with an appropriate amount of viscosity. The macromolecule protein produces IgG, including IgG1 and IgG2, and its immunization efficacy is tested in a murine model. 1,2-dimyristoyl-sn-glycero-3 ethyl-phosphocholine (EDMPC) and linoleic acid (Lin) are the cationic and anionic components of the ionic liquid. Yucatan micropig skin comprising 100 μm-thick patches were used for the in vitro permeation testing; the hydrophobicity of the IL-S/O formulation enhanced the drug delivery into the skin. Furthermore, the in vivo immunization studies were carried out with C57BL/6NJc1, and after 56 days of immunization with OVA, there was an increase in the IgG titers of the IL S/O patch, confirming that the production of anti OVA IgG was due to the OVA presenting itself to APCs. All the results indicate the significance of IL-S/O patches and their potential in delivering the antigens through the transdermal route [80].

Apart from these, ionic liquids were also used as nanocarriers with an attached alkyl chain varying in length to form surface-active ionic liquids (SAILS) [81]. They act by assembling themselves into the vesicular and micellar nanocarriers of the aqueous solution. The current study evaluated the synergistic effect of the complete transmembrane (TM) sequence of ErbB2 protein in combination with the novel SAILs to determine their anticancer activity. SAILs were designed for improving permeability and transporting the transmembrane peptides to the targeted pathological site. 4-dimethylaminopyridine and thionyl chloride underwent esterification in order to develop the mono-amphiphilic ionic liquid carrier ProCl; for the second one, ester and SLS underwent metathesis in hot water in order to develop the Bi amphiphilic carrier ProLS. The BT474, AU565, A549, and CFPAC-1 cell lines were studied for the specificity of the transmembrane peptides and the transmembrane peptide nanocarriers; all the chosen cell lines have high ERBB2 RNA expression. Bi-amphiphilic carriers have shown a 20–50% inhibitory effect against AU565 cells and a decrease in cell viability as their concentrations increase. A synergistic activity between the V3 transmembrane peptide and the bi-amphiphilic carrier resulted in high anticancer activity against all the ErbB2-positive cell lines that were used by inhibiting the ErbB2 receptor dimerization. This led to successful ErbB2 downstream signaling, especially within the MAPK and PI3K-At pathways, which was indicated by a drastic reduction in phosphorylation levels of Erk1/2 and Akt, respectively. The high cytotoxic effect on the cell lines clearly explains the synergistic effect of SAIL and TM peptides, which is better than the TM peptide and SAIL carrier alone [82]. The safety profiles and toxicity of ionic liquids are reported in Table 2.

## 6. Ionic Liquids as Vaccine Adjuvants

Vaccine adjuvants play a crucial role in enhancing the immune response to vaccines. Adjuvants are substances added to vaccines to enhance the body’s immune response to the provided antigens. When combined with vaccines, adjuvants work in several ways: (1) Adjuvants stimulate a stronger and more prolonged immune response to antigens. They themselves can activate various immune cells, such as dendritic cells, which are crucial for initiating the immune response. (2) By activating immune cells, adjuvants improve the presentation of antigens to T cells and B cells, which ensures that the immune system recognizes and responds more effectively to the vaccine antigens. (3) Adjuvants can boost the production of antibodies by B cells. (4) Adjuvants help in the formation of memory cells, which “remember” the antigens. (5) Adjuvants also protect the antigen from biodegradation, thus increasing its stability. (6) Adjuvants also reduce the amount of antigen needed in the vaccine [87].

Currently, there are only a few adjuvants available in the market for vaccine formulation development. Among the most common marketed adjuvants, MF59 and AS03 are oil-in-water emulsions used in influenza vaccines, helping to stimulate a stronger immune response and reduce the amount of antigen needed. Another adjuvant, AS04, a combination of alum and a toll-like receptor 4 (TLR4) agonist, is used in HPV and hepatitis B vaccines to enhance the immune response. CpG 1018, a synthetic DNA sequence that mimics bacterial DNA, is used in some hepatitis B vaccines to stimulate a robust immune response [88]. Adjuvants can be classified according to their physicochemical properties, origin, and mechanisms of action [89]. Based on their mechanisms of action, adjuvants can be divided into delivery systems (particulate) and immune potentiators (immunostimulatory) [90]. Currently there are only six marketed adjuvants approved by the Food and Drug Administration (FDA) as a part of vaccines in the United States. Even the FDA-approved vaccine adjuvants are not licensed individually, and they are only approved as a part of the vaccines. The properties and limitations of these are shown in Table 2. Besides these marketed products, there are plenty of adjuvants in the research phase. Most of these are emulsions, small molecules, or combination adjuvants. These all are in different phases of human trials. The NIH has listed 91 prospective adjuvants in a vaccine adjuvant compendium which will help the vaccine developers to select the appropriate adjuvants [91].

Adjuvants increase the immune responses to an antigen when combined with vaccines; however, these adjuvants tend to undergo denaturation. To overcome this, the stabilization effects possessed by the ILs will aid in vaccine formulations as adjuvants. With the straightforward synthesis of ILs and their high tunability, ILs are considered as a new class of adjuvants [92,93].

Due to lack of understanding about the mechanisms and immune effectiveness of the adjuvants, there is difficulty in matching appropriate adjuvants for vaccines [54]. Over the past decade, one of the advancements that helped the researchers to gather an in-depth understanding of the mechanism of action of the adjuvants was to develop better ones using systems biology. This is not only used to comprehend the adaptive and innate responses that are driven by molecular networks and gain insights on the initiation and control of immune responses but also to interpret the molecular signatures in order to hypothesize the vaccine’s efficacy and provide mechanistic insights [16]. There are several mechanisms through which the adjuvants tend to enhance the immune responses generated by the vaccines. Generally, adjuvants improve the activation of antigen-presenting cells (APCs) through antigen-presentation signals and costimulatory signals. It is known that the activation of pattern recognition receptors (PRRs) on APCs can be achieved through immunostimulants—a category of adjuvants which lead to increased cytokine upregulation. They also act by activating the dendritic cells and through modification of the cytokine profile [54].

Upon comparison with other anionic ILs, Choline and Sorbic acid (ChoSorb) was evaluated for its potential as an adjuvant using the model protein antigen ovalbumin (OVA) and a recombinant hemagglutinin protein (rHA) at low concentrations. When intramuscularly administered in BALB/c mice as a single dose, ChoSorb with OVA has successfully induced the anti-OVA IgG antibodies, thus enhancing the antibody response and proving itself to be an excellent adjuvant candidate. The responses in the ChoSorb group were higher than those when compared with OVA alone and OVA with alum, a conventional adjuvant. Apart from this, ChoSorb OVA vaccination has also resulted in a significant increase in Germinal center (GC) formation; this formation is typically located in the lymph nodes where B-Cells are activated and matured, thereby producing long-lasting antibodies. This is an indication that ChoSorb promotes the humoral immune response that is essential for long-term immunity. In a C57BL/6 prime boost immunization model, ChoSorb has significantly increased the levels of IL-6 and OVA-specific CD8+ T-cells (after seven days of a booster dose), better initiating immune responses compared to other ILs and both the control groups. And after fourteen days from the booster dose, ChoSorb enhanced both the Th-1 and Th-2 type responses. Achieving both responses from a single adjuvant is exceptional as more than half of the adjuvants need two separate immunomodulatory components for achieving both cellular and humoral responses, hence proving that ChoSorb is the first IL adjuvant capable of achieving both the responses [94].

Apart from this, Choline and lactic acid were synthesized in a molar ratio of 1:2, and the formed product ChoLa was used as an adjuvant with OVA and was compared with alum as suggested by the literature due to alum’s establishment as a gold standard [95]. Compared to alum, ChoLa has a five times higher antigen dispersion of OVA in ex vivo porcine skin than alum, allowing it to have greater antigen presentation for immune cells [96]. This newly synthesized IL has also been shown to result in a significant increase in the CD86 expression of dendritic cells along with a 25% increase in CD4+ T cell infiltration, proving an improved local immune response at the injection site. An adjuvant is required to activate the innate immune cells, and ChoLa has successfully demonstrated this potential. Differing from alum’s Th2-biased immune response, this IL adjuvant has shown a Th-1 response with a 5-fold increase in CD8+ cells when compared to controls [97].

In another study, a novel IL-based nano emulsion was used for intranasal delivery against the influenza split virus antigen. Choline and Niacin (ChoNic) is the IL that was formulated with squalene and Tween 80. The formulation also used influenza split virus as the antigen to evaluate the nasal immune effects of using an IL as an adjuvant. The IL’s stability has been proven with the exceptional uniformity of this nano emulsion after having been stored for 12 months; with the particle size only increasing from 168.6 to196.5 nm. However, the oil-in-water emulsion without the IL showed an increase in size to 1006 nm over the same period. Moreover, 6–8-week-old BALB/c mice were intranasally immunized twice either with PBS as a control group, the IL nano emulsion, or the commercial oil-in-water MF59 emulsion. Antigen-specific sIgA levels in the nasal lotion were measured on day 28 using ELISA to determine the local production and secretion of antigen-specific secretory IgA antibodies as they play a major role in the mucosal adaptive immune response [98]. The IL nano emulsion has shown an exceptionally high section of sIgA-specific antibodies from nasal mucosa, having 25-fold more titers than PBS and 5.8-fold more titers than the MF59 emulsion. It has been established that the mucosal responses are from the prolonged retention of antigens, making ChoNic a great prospect as a novel adjuvant [99].

Apart from using ionic liquids as direct adjuvants, stabilization studies of ILs on antigens in nano formulations, using 1-dodecyl-3-methylimidazolium bis(trifluoromethyl sulfonyl)amide, in an ionic liquid-based solid-in-oil (S/O) nano dispersion, have shown significant increases in the antigen-specific antibody levels without requiring immune stimulation from adjuvants. The authors formulated the S/O nano dispersions using isopropyl myristate combined with two different surfactants: sucrose laurate (L-195) and sucrose eructate (ER-290). An in vitro release study of these nanoparticles conducted in PBS solution validated the benefits of incorporating IL in formulations. Several benefits, including the sustained release of the model antigen (ovalbumin, OVA), prevention of premature degradation, and enhanced interface stabilization by maintaining the structure of the nanoparticles without allowing them to break ahead of time, were observed. Upon transcutaneous administration in BALB/c mice, OVA-specific IgG titers were measured; ELISA results have confirmed the higher levels of OVA-specific serum IgG in mice that were administered with S/O nano dispersions containing ILs compared to those with the PBS control and formulations without IL. Moreover, the toxicity of ILs was also monitored in the mice, and no significant weight changes were observed [6].

Similarly, lipid-based ionic liquids (LBILs) that were previously used as solubilizing materials were used for enhancing anticancer effects. In this study, EDMPC LIN was synthesized with OVA protein and Tween 80 along with isopropyl myristate as a protein-containing nanocarrier (PCNC). The immunotherapeutic responses to the PCNCs were studied in C57BL/6N mice. Plasma was evaluated for the antitumor antibodies of IgG1 and IgG2a, and the tumor microenvironment was studied for the expression of CD8+ T cells. To determine the IgG-specific antibodies in the mice, the IL-based PCNC solution was applied on the skin for 24 h for 3 weeks in 1-week intervals, and antibody levels were determined using ELISA. PBS with OVA was used as the control group, and blood collections were conducted before immunizations and after anesthetizing the mice on the third week. The IL-based formulation resulted in a more significant increase in the IgG1 and IgG2a levels than the aqueous and CPE-based formulations. Although, there were similar TH2 and TH1 immune responses from the IL-based formulation and the other injections; finetuning the Th1/Th2 balance can be used towards treating autoimmune diseases. These results show that the IL-based PCNC possess a strong immunotherapeutic anticancer effect and can also inhibit tumor growth without toxicity [100]. The antigens and properties of the used ionic liquids from the above studies are mentioned in Table 3.

Based on the above results, ILs have proven their ability as novel vaccine adjuvants prompting immune responses better than marketed adjuvants. Further studies can be undertaken to compare them with regular adjuvants and estimate their toxicity.

## 7. Conclusions

Although ionic liquids (ILs) have been used in the chemical industries as a potential replacement for traditional volatile and harmful organic solvents for many purposes, they do have some limitations.

Additionally, the use of ionic liquids in pharmaceuticals and other sensitive applications may face regulatory hurdles due to the need for extensive safety and efficacy testing. Many ionic liquids are not readily biodegradable, which can lead to environmental concerns if they are released into the environment. While some ionic liquids have high viscosity, this may affect their handling and processing in industrial applications. Moreover, there are still limited data on the long-term effects and behavior of ionic liquids in various applications, which can hinder their application. Although ILs have been widely used in other fields, they are relatively new in the pharmaceutical industry. Due to their malleable physical and chemical properties, ionic liquids can be potential candidates to be used as excipients. They can be used in drug dosage forms to increase solubility, drug permeability, and stability, to provide a charge to drug formulations, and for site-specific or organ-targeted delivery. With their diverse properties, using ionic liquids alone can replace several excipients in drug formulations, making them more efficient and less costly. Another application of ILs is in vaccine formulations; currently, there is an urgent need for developing new adjuvants that can address these issues in vaccine formulations. The safety, solubility, flexibility, and tunable properties of ionic liquids make them promising candidates as adjuvants. The potential of ionic liquids (ILs) as vaccine adjuvants is an exciting area of research due to their unique properties. ILs can enhance the immune response by improving the delivery and presentation of antigens to the immune system. They can stabilize vaccine components, such as proteins and peptides, protecting them from degradation.

As discussed, ILs can also be used in formulations for the controlled release of antigens, ensuring a steady and prolonged immune response. The unique properties of ILs can be easily adjusted by altering the cations and anions, allowing for the customization of adjuvants to meet specific needs. All these properties make them versatile candidates for different types of vaccines. Developing new vaccine adjuvants is a high priority now, as there are only a few marketed adjuvants available to fulfill the requirements of all upcoming vaccines. Therefore, ionic liquids can open a new window to explore greener, more effective, and low-cost adjuvants.

## Figures and Tables

**Table 1:** Structural formals for ionic liquids.

IONIC LIQUIDS	STRUCTURAL FORMULAS
ChoSorb—Choline, Sorbic Acid	CH_3_ | CH_3_-N^+^-CH_2_-CH_2_-OH | CH_3_ + CH_3_-CH=CH-CH=CH-COOH
ChoLa—Choline and lactic acid	CH_3_ | CH_3_-N^+^-CH_2_-CH_2_-OH | CH_3_ + OH | CH_3_-CH-COOH
ChoNic—Choline and Niacin	CH_3_ | CH_3_-N^+^-CH_2_-CH_2_-OH | CH_3_ + COO- | C_5_H_4_N
1-dodecyl-3-methyl imidazolium bis(trifluoromethyl sulfonyl) amide	N / \ C_12_H_25_ CH_3_ \ / N | O + O O || || CF3-S-N-S-CF3 || || O O
EDMPC LIN-1,2-dimyristoyl-sn-glycero-3-ethyl-phosphatidylcholine, Linoleic acid	CH_2_-O-CO-(CH_2_)_4_CH=CHCH_2_CH=CH(CH_2_)_7_CH_3_ | CH-O-CO-(CH_2_)_12_-CH_3_ | CH_2_-O-P-O-CH_2_-CH_3_ | O^−^ | CH_2_-CH_2_-N+(CH_3_)_3_
[BMIM][Ac]-1-Butyl-3-methylimidazolium acetate	N / \ C_4_H_9_ CH_3_ \ / N + CH_3_-COO^−^
[Chol][Ac]—Choline acetate	CH_3_ | CH_3_-N+-CH_2_CH_2_OH | CH_3_ + CH_3_-COO-
[Chol][Prop]—Choline propionate	CH_3_ | CH_3_-N+-CH_2_CH_2_OH | CH_3_ + C_2_H_5_-COO^−^

**Table 2:** Adjuvants in the US market approved by the FDA as a part of vaccines with their properties and limitations.

NAME OF MARKETED ADJUVANT	PROPERTIES	LIMITATIONS	VACCINES	REFERENCES
Aluminium Hydroxide, Aluminium Phosphate	Enhances production of IgG1 and IgE by enhancing Th2 responses. Prolongs bioavailability. Enhances antigen presentation.	Possible allergic reactions and weak cellular immune response.	DTaP, Pneumococcal conjugate vaccine, HPV vaccines, haemophilus Influenza type b, hepatitis A, hepatitis B	[83,84,85,86]
Potassium Aluminum Sulphate	Enhances immune responses. Stabilizes vaccines and creates a depot for slow antigen release.	Replaced by aluminium hydroxide and aluminum phosphate due to poor reproducibility and poor Th1 responses.	Decavac-Td Vaccine, DT vaccine-Diptheria and tetanus vaccine.	
MF59	Immune stimulant. Improves antigen delivery.	Biased Th2 immune response and weak Th1 response.	Fluad	
AS01—Monophosphoryl Lipid A+ QS21	Boosts antigen signals on APCs. Immunostimulatory effects. Contains alpha tocopherol as an immunostimulant.	Similar to MF59; shows weak Th1 response.	Shingrix	
AS04—Monophosphoryl Lipid A + aluminium salt	Stronger immunostimulatory effect than alum. Contains monophosphoryl Lipid A as immunostimulatory molecule. Used to elevate antibody production.	Weaker cellular immunity, especially CD8+ T cell responses.	HPV	
CpG 1018	Produces pro-inflammatory cytokines. Prompts stronger Th1 responses.	The addition of alum is necessary for enhancing their effect.	Heplisav-B	
Matrix M	Stimulates immune system.	Limited long-term data available.	Novavax adjuvanted-COVID-19	

**Table 3:** Ionic liquids and their properties.

IONIC LIQUID USED	ANTIGEN	SAFETY PROFILE	PROPERTIES OF IONIC LIQUID	REFERENCES
ChoSorb—Choline and Sorbic Acid	Ovalbumin, SARS-CoV-2 Spike protein	Used materials generally regarded as safe for IL synthesis. Low risk of toxicity due to addition of adjuvant.	Produced both cellular and humoral responses; effective in low doses.	[94]
ChoLa—Choline and lactic acid	Ovalbumin	Natural metabolites in human body classified as GRAS. Potential toxicity will be reported in future studies.	Potent immune response against the antigen; distributed antigen effectively.	[96]
ChoNic—Choline and Niacin	Influenza split virus antigen	In range of physiological salts—IC50 was 57.63 ± 5.63 mmol L^−1^.	Enhanced humoral response. The oil in IL used as injection adjuvant induced stronger humoral responses than MF59.	[99]
1-dodecyl-3-methyl imidazolium bis(trifluoromethyl sulfonyl) amide	Ovalbumin as model antigen	Does not specifically mention toxicity, as ILs are green solvents which suggest a favorable safety profile.	Enhanced skin permeability along with increased OVA-specific serum IgG compared to control and non-IL formulation.	[6]
1,2-dimyristoyl-sn-glycero-3-ethyl-phosphatidylcholine, Linoleic acid- EDMPC LIN	Ovalbumin as model antigen	Tested in animal model; no adverse effects observed.	Simulated OVA-specific tumor response suppressed tumor growth and improved survival rates. Increased cytotoxic T cells expressing CD8 antibodies in tumor microenvironment.	[100]
ChoNic—Choline and Niacin	Inactivated foot and mouth disease virus (iFMDV)	Has not been discussed specifically, as both compounds are naturally occurring metabolites.	Improved antigen dispersion and humoral responses when compared to unadjuvanted iFMDV.	[78]
ChoH_2_PO_4_ ChoCl ChoSO_4_	Inactivated foot and mouth disease virus (iFMDV)	Has not been discussed specifically, considering that Choline is regarded as safe by the FDA.	ILs did not affect the immunogenicity of iFMDV antigens but have outperformed other stabilizers like BSA and sorbitol.	[79]

## Data Availability

Not applicable.
